# Assessment of Product Variety Complexity

**DOI:** 10.3390/e25010119

**Published:** 2023-01-06

**Authors:** Vladimir Modrak, Zuzana Soltysova

**Affiliations:** Faculty of Manufacturing Technologies, Technical University of Kosice, Bayerova 1, 080 01 Presov, Slovakia

**Keywords:** complexity, mass customization, product variety, entropy, product configurations

## Abstract

Product variety complexity assessment plays a vital role in system design, as it has tremendous negative effects on manufacturing complexity in assembly systems and supply chains. On the other hand, practitioners and researchers frequently consider the number of product variants as a sufficient measure to be used to manage this kind of complexity. However, as shown in this study, such a measure does not reflect all pertinent features of complexity. Therefore, the main goal of the paper is to develop a measurement method for product variety complexity that adequately reflects relevant relations between the portfolio of optional components and the number of product variants. As presented in the paper, the concept of information theory can be effectively applied to measure product variety complexity. Moreover, such a measure can also be useful to better understand this system’s properties in order to reduce the level of variety-induced complexity. As such, the proposed method can be viewed as a complementary tool for reducing manufacturing complexity in terms of mass customization. The developed complexity metric was successfully tested on a realistic design example.

## 1. Introduction

In the past few decades, one can witness a considerable development in research addressing the mass customization of production. This global trend naturally resulted in more complex products and manufacturing processes to produce them. Even though product and process complexity are inherent and cannot be avoided, highly customized products have to be kept affordable and sustainable. On the manufacturer’s side, the reason why is quite clear. As research studies show, increased product variety in the automobile industry creates more complexity, as a result of which there is an increase in the total costs of up to 20% [1]. Moreover, Hichert [2] found out that a significant increase in the total costs of the electrical appliances was caused due to product variety. In other words, customer requirements in a mass customization environment push manufacturers to broaden their product variety, which increases at least manufacturing complexity and organizational complexity, and as such, a company’s internal complexities negatively affect their total costs [3]. In this context, Child et al. [4] are of the opinion that a company should evaluate the level of product variety at which consumers will still be satisfied and the level of complexity that will keep the company’s costs down. 

According to Fisher et al. [5] product variety can be defined in two ways: (1) As “the breadth of products that a firm offers at a given time and the rate at which the firm replaces existing products with new products”. Product variety is here referred to according to the first mentioned definition, and then (2), logically, one could interpret the term product variety complexity, taking an internal company’s view, as complexity driven by the number of end product variants that the manufacturer offers to customers [6]. Intuitively, practitioners and researchers take this information, i.e., the maximum number of possible product variants (PPVs), as a measure to quantify product variety complexity (see, e.g., [7,8]). However, complexity in general is the science of studying the phenomena that emerge from a collection of interacting objects [9] and requires continuous longitudinal research [10]. Before introducing a proposed product variety complexity construct for this specific purpose, it can be useful to present a very brief view on product complexity as such. By paraphrasing the author of [11] on this issue, he states that if complex products are tangible things, then complexity and product are synonymous, where product is prime. In other words, a product’s utility is more important than the potential risks associated with its complexity. In this context, the presented work aims to reveal some important disadvantages of the product variety complexity measurement based on the number of possible product variants and to propose a more reliable method to measure it.

The main contribution of this study lies in the development of the new, unique solution to quantify variety-induced complexity. The method is based on the transformation of a product platform structure consisting of a wide portfolio of modules or components and related product configurations, i.e., PPVs, into a design structure matrix (DSM), which unequivocally reflects the mutual relation between functional requirements (FRs) and design parameters (DPs). The most significant initial step in this effort was to formalize the mutual relation between product platform components (PPCs) and PPVs. It was proposed to display it in the form of a bipartite graph, in which PPCs represent DPs and PPVs are rightfully considered FRs. After that, for any such bipartite graph representing the relationship between FRs and DPs, DSM can be defined. Subsequently, we applied one of the possible methods for structural complexity estimation, which assumes that the distribution of FR-DP couplings adequately reflects the complexity of a design solution. As will be shown further on in Section 3 and Section 4, the proposed method offers an effective and reliable way to quantify product variety-induced complexity that can be easily implemented in practice. To substantiate this assertion, much of the paper’s content provides research-based evidence that the proposed method estimates variety-induced complexity more adequately than a number of PPVs. As evidence-based practice is an equally important source for the validation and verification of research processes and output, a practical case study is also used to support the above-mentioned assertion.

## 2. Related Work

Variety-induced complexity, as a term, can be comprehended as an internal attribute of a product affected by both internal and external influences [12]. As is already known, mass customization brings with it an extensive product variety, which is associated with high complexity, especially in final assembly operations [13]. In this context, the extent of product variety is considered one of the most persistent problems in mass customization practice [14]. The existing literature offers several complexity metrics in terms of product variety. Ericsson and Erixon [15] proposed the so-called *interface complexity* metric, which is based on the assumption that this kind of complexity is low when minimum information flows between design modules are needed. Abdelkafi [16] consistently analyzes the development stages of metrics for the evaluation of product platforms. It is worth mentioning that the authors of [17,18] developed several useful metrics for the evaluation of platform-based product families. According to Brecher [19], a possible indicator for the estimation of product variety complexity is the number of component variants in the product’s structure. Moseley et al. [20] presented a comprehensive literature review on variety, complexity, and manufacturing operational performance. They showed that product variety has a consistently negative influence on manufacturing operational performance. ElMaraghy and ElMaraghy [21] proposed several related concepts and a multi-faceted strategy for managing variety-induced complexity. According to their view, management of variety-based complexity might consider product, process, and market to be the key elements that are critical in designing a marketing and manufacturing strategy for a company. In this context, some new process complexity measurement methods were recently developed. One of them, the Process Complexity Index [22], is based on the difference in variety within the identified process complexity drivers. Several relate to process metrics and were also presented in the works of [23,24], which adopt the concept of Shannon’s information entropy.

The above-mentioned research clearly demonstrates that the increased complexity is directly proportional to both the number of variable components and the number of product variants. Naturally, approaches to overcome this problem vary depending on the theoretical models, preferred methodologies, parameters used, and specific research objectives. For instance, Zhu [25] reviews the complexity model developed for mixed-model assembly lines and demonstrates the opportunity of minimizing complexity through assembly sequence planning. In his model, he considers the product variety-induced complexity in assembly lines, where operators are making choices according to the variety of parts, tools, fixtures, and assembly procedures. Efthymiou et al. [26] analyze in their paper several methods and approaches pertaining to the research of manufacturing systems’ complexity. The authors also proposed definitions of complexity types and identified a taxonomy of complexity analysis methods in the same paper. Rennpferdt et al. [27] investigated and showed how a design for variety approach can be linked with a complexity cost. Martínez-Olvera [28] developed a discrete-event simulation model to obtain data for an entropy-based metric that addresses both the mass customization variety-induced complexity and the complexity derived from the adoption of the Industry 4.0 paradigm. It is also important to mention that prevalent approaches toward measuring system complexity (see, e.g., [29,30,31]) are based on the information theory fundamentally developed by Shannon and Weaver [32]. In this context, Blecker and Abdelkafi [33] defined variety-induced complexity from the perspective of the company. This work also corresponds with the previously conducted research presented in the work by the authors of [34]. There are also several related works using DSM for measuring the structural complexity of systems. Wang et al. [35] identified the complexity of the engineering structures in large-scale engineering design by using a DSM matrix. The authors of [36] claimed in their work that DSM can be helpful in reducing an organization’s complexity. Braun and Lindemann [37] used a DSM matrix to investigate the influence of structural complexity on product costs. A framework to manage and optimize system complexity was presented in the work of the authors of [38]. Other applications of DSM in the context of complexity measurement and management have been discussed in the works of [39,40,41,42]. Accordingly, it can be stated that the application of a DSM can have a positive impact on complexity management by focusing attention on the elements of a complex system and their relationships to each other.

Obviously, it is not possible to provide a complete set of references on this topic since similar research is branching in a number of directions.

## 3. Proposed Product Variety Complexity Measure

### 3.1. Theoretical Basis

The main idea of the approach proposed here is to apply the structural design complexity measure, which is based on the Boltzmann-Gibbs entropy concept to the quantum realm [43]. Given that entropy is often associated with the amount of disorder in a thermodynamic system, the more disordered a system is, the more information is needed to describe it, and thus the higher the entropy. In statistical mechanics, the disorder in the distribution of the system over the possible microstates is a subject of study [44]. Boltzmann was the first to propose a method for calculating the entropy using the formula:

(1) $$S_{B}=k_{B}\cdot \ln\left(W\right)\;\;\left[Joule/Kelvin\right],$$

where *k_B_* is a thermodynamic unit of measurement of entropy and is known as the Boltzmann constant and “*W*” is the degree of disorder.

Quantity “*W*” can be counted using the formula:

(2) $$W=\frac{V!}{V_{1}!\cdot V_{2}!\cdot \ldots \cdot V_{i}!}\;,$$

where *V* is the number of particles in an ideal gas of negligible volume, while “*i*” ranges over all possible molecular microstates. If we set the Boltzmann constant to 1, without loss of generality, then entropy is expressed as:

(3) $$S_{B}=\ln\left(W\right),$$

this form of entropy corresponds to Shannon’s entropy, which measures the amount of information in natural units—nats.

Even though a statistical entropy perspective was officially introduced in 1870, Boltzmann a few years earlier probably intuitively used the following formula [45]:

(4) $$S_{B}=\rho \cdot \ln\rho ,$$

where *ρ* is interpreted as a density in phase space, but implicitly it corresponds to a statistical entropy. Later, Gibbs generalized statistical entropy for thermodynamic systems, in which the microstates of the particles may not have equal probabilities “*p_i_*” using the formula:

(5) $$S_{G}=k_{B}\cdot \sum^{\;}p_{i}\cdot lnp_{i}.$$

The concept of entropy inspired many new disciplines, including design science (see, e.g., [46,47,48]), which is the subject of this article. As is already known, advanced design science techniques are often focused on design space generation and exploration [49]. As design space represents the domain of possible designs given the constraints and variables, it can be formalized by DSM, which shows the relationships between elements in a system. Moreover, DSM is quite often used as a tool for managing complexity [50,51]. In this context, Guenov [52] showed that there are sufficient analogies between the degree of disorder in a system and the information entropy of design space. He hypothesized that the relationship between a distribution of FRs and DPs gives a good idea of complexity. Based on the mentioned analogies and in accordance with the Boltzmann function for a statistical description of the quantum system, he proposed the following formula to quantify structural design complexity:

(6) $$S_{DC}=\sum^{\;}Nj\cdot lnNj\;[\mathrm{nats}],$$

where *Nj* is the number of couplings per design parameter (i.e., per column), and *j = 1,…, K*.

An application demonstration of Equation (6) on a simple design matrix (DM) with its coupling distribution is shown in the following matrix.

$$\left[\begin{array}{c} FR_{1}\\ FR_{2}\\ FR_{3}\\ FR_{4}\\ FR_{5} \end{array}\right]=\left[\begin{array}{ccccc} X & 0 & 0 & 0 & 0\\ 0 & X & 0 & 0 & 0\\ 0 & X & X & 0 & 0\\ 0 & 0 & 0 & X & 0\\ 0 & X & X & 0 & X \end{array}\right]\left[\begin{array}{c} DP_{1}\\ DP_{2}\\ DP_{3}\\ DP_{4}\\ DP_{5} \end{array}\right]$$

then structural design complexity is calculated as follows:

$$S_{DC}=1\cdot ln1+3\cdot ln3+2\cdot ln2+1\cdot ln1+1\cdot ln1=4.68\;nats.$$

### 3.2. Adaptation of Structural Design Complexity to Product Variety Complexity

In order to adapt Equation (6) for quantification of product variety complexity, the following considerations were taken into account:

(i) Platform for a customized product in a simplified manner usually consists of three basic types of components, i.e., stable (S), optional (O), and compulsory optional (CO), while CO are limited in selection. The limits can be specified by at least three types of volitional rules: minimum, maximum, and particular requirements on selection. Then, one can determine all PPVs using specific combinatorial constructions, as is demonstrated below. Let us denote a group of cases that have the same number of stable components as class *CL_i_*_,_ where *i* = 1–∞, and a group of cases that have the same number of optional components as sub-class *SCL_j_*_,_ where *j* = 0–∞. Then, PPVs can be identified as follows: when the structure of product configurations is unimportant, the formula is:
  (7) $$CL_{i}SCL_{j}=2^{j},$$
  and when the structure of product configurations is important, the formula is:
  (8) $$CL_{i}SCL_{j}=\sum_{j=0}^{n}\left(\frac{n!}{j!\left(n-j\right)!}\right),$$

where *j* is a set of integers limited to the range from 0 to *n*.

**Figure 1:**
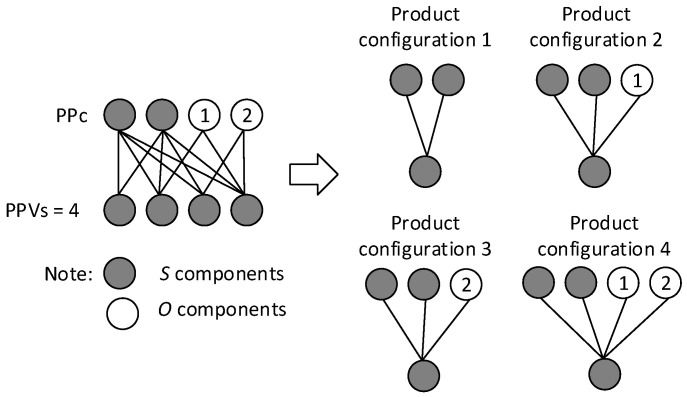
An example of a product platform with two stable and two optional components.

Let us have an example of a product platform with two stable components and two optional components. In the case where a distribution of product configurations is important, Formula (8) can be applied:

$$CL_{2}SCL_{2}=\left(\frac{2!}{0!\left(2-0\right)!}\right)+\left(\frac{2!}{1!\left(2-1\right)!}\right)+\left(\frac{2!}{2!\left(2-2\right)!}\right)=4$$

then it is possible to create four product configurations (see Figure 1) where the relationships between PPc and PPVs are represented by bipartite graphs.

All PPVs based on the number of S, O, and CO components can be calculated using the following formula:

(9) $$CL_{i}SCL_{j}^{k}=\sum_{j=0}^{n}\left(\frac{n!}{j!\left(n-j\right)!}\right)\cdot \sum_{l=1}^{k}\left(\frac{k!}{l!\left(k-l\right)!}\right),$$

where *k* is the number of all possible CO components and *l* is defined as the number of components to be selected from *k* components.

**Figure 2:**
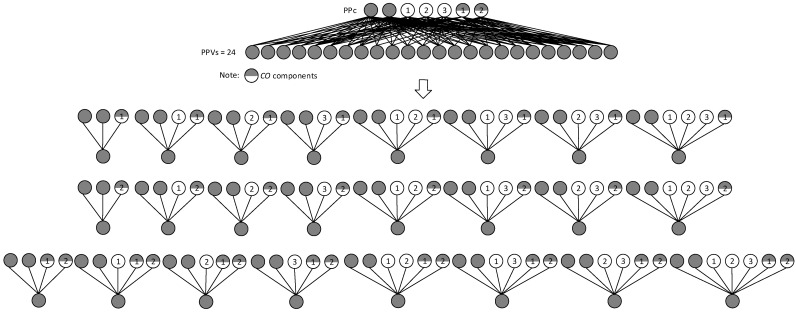
An example of a product platform with two stable, three optional, and two compulsory optional components.

**Figure 3:**
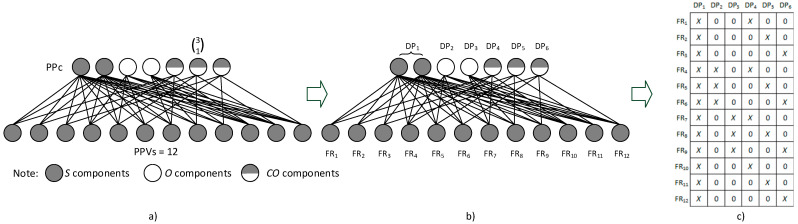
(**a**) An example of mutual relationships between PPc and PPVs; (**b**) related relations between DPs and FRs; and (**c**) a related design matrix.

For example, when we have a PPc with two *S* components, three *O* components, and two *CO* components (applying the rule of choosing the minimum of one component out of two), then the number of PPVs can be enumerated by Formula (9) as follows:

$$CL_{2}SCL_{3}=[(\frac{3!}{0!\left(3-0\right)!})+\left(\frac{3!}{1!\left(3-1\right)!}\right)+\left(\frac{3!}{2!\left(3-2\right)!}\right)+\left(\frac{3!}{3!\left(3-3\right)!}\right)]\cdot [\left(\frac{2!}{1!\left(2-1\right)!}\right)+\left(\frac{2!}{2!\left(2-2\right)!}\right)]=8\cdot 3=24$$

based on the obtained results the number of PPVs = 24. All the product configuration structures are graphically presented in Figure 2;

(ii) Further, it is assumed that the product platform components reflect in reality product design parameters, and that PPVs represent functional requirements specified by individual customers. Then, the relationship between PPc and PPVs can be substituted by the relationship between FRs and DPs (see Figure 3a,b). As the number of S components does not impact PPVs, it is represented by only one DP;
(iii) Subsequently, couplings between FRs and DPs are described through a design matrix (see Figure 3c).

After that, Equation (6) can be applied to enumerate product variety complexity as follows:

$$S_{DC}=12\cdot \ln12+3\cdot \ln3+3\cdot \ln3+4\cdot \ln4+4\cdot \ln4+4\cdot \ln4=106.1\;\mathrm{nats}$$

when applying Equation (9), PPVs are enumerated as follows:

$$CL_{2}SCL_{2}^{3}=\left[\left(\frac{2!}{0!\left(2-0\right)!}\right)+\left(\frac{2!}{1!\left(2-1\right)!}\right)+\left(\frac{2!}{2!\left(2-2\right)!}\right)\right]\cdot \left[\left(\frac{3!}{1!\left(3-1\right)!}\right)\right]=4\cdot 3=12$$

a possible usefulness of this product variety complexity measure will be briefly explored in the next sub-section.

### 3.3. Comparison of S_DC_ against PPVs

**Figure 4:**
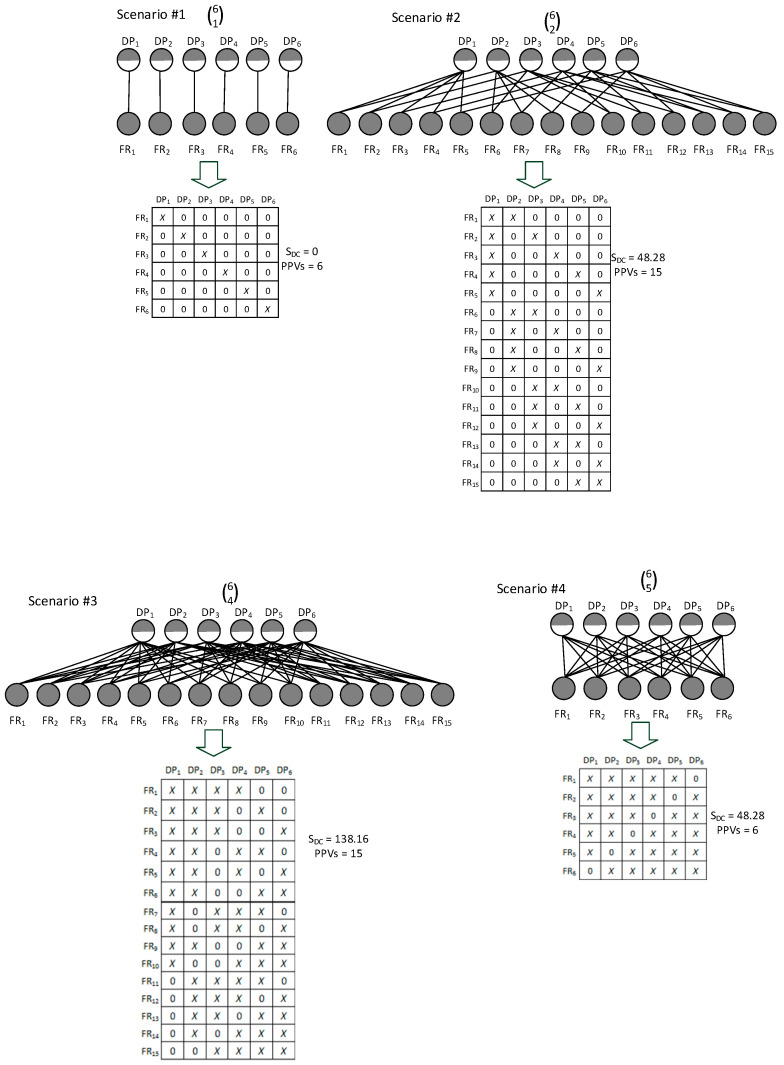
An example of a scenario with six CO components and all possible individual selectivity rules.

In order to analyze possible differences between the two product variety complexity measures, i.e., S_DC_ and PPVs, the following computational experiment on a selected PPc will be used. Let us say that PPc consists of six CO components. Further, four different individual selectivity rules $\left(\begin{array}{c} 6\\ 1 \end{array}\right)$,$\;\left(\begin{array}{c} 6\\ 2 \end{array}\right)$,$\;\left(\begin{array}{c} 6\\ 4 \end{array}\right)$, and $\left(\begin{array}{c} 6\\ 5 \end{array}\right)$ will be gradually employed in order to map differences between PPVs and S_DCs_. Subsequently, the obtained PPVs and S_DC_ values from these four scenarios (see Figure 4) will be compared.

Based on the computational results depicted in Figure 4, the following substantial differences between PPVs and S_DCs_ were identified.

Let us compare scenario #1 with scenario #4 and scenario #2 with scenario #3, since these pairs of scenarios consist of an identical number of PPVs. Starting with the first pair of scenarios #1 and #4, one can see that in scenario #1 the related DM is uncoupled, while in scenario #4 it is heavily coupled. As a consequence, the S_DC_ of scenario #1 equals 0 nats, and the S_DC_ of scenario #4 is 48.28 nats. These differences provide clear evidence that S_DC_ reflects product variety complexity of these two PPc more realistically than PPVs.

Similarly, the other two scenarios, #2 and #3, can be compared. In both cases, the DMs are coupled. However, the design related to scenario #2 is less coupled than the design in the case of scenario #3. Assuming in general that a less coupled design is less complex than a more coupled one [53] and that the ideal composition of FRs and DPs is considered the simplest one [52], it is possible to say that the variety-based complexity of the DM related to scenario #2 is less complex than the DM of scenario #3. This argumentation corresponds to the values obtained by the indicator S_DC_, but based on the numbers of PPVs, both designs are equally complex.

Thus, S_DC_ can be considered a more suitable option to measure product variety complexity than the number of PPVs. The main difference between PPVs and S_DCs_ lies in the fact that S_DC_ inherently incorporates important principles of system complexity metrics [54], i.e., the complexity of a system scales with:

- The number of its elements;
- The number of interactions between the elements;
- The complexities of the elements;
- The complexities of the interactions.

## 4. Practical Case Study

**Figure 5:**
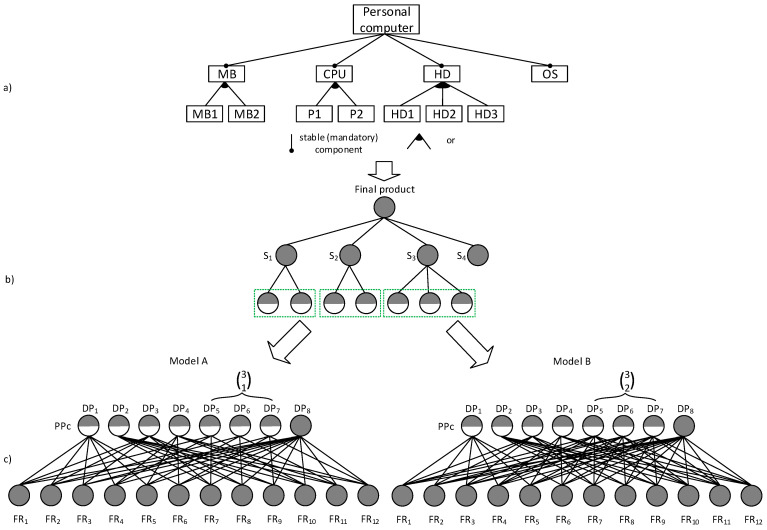
(**a**) Feature diagram; (**b**) Transformation of the feature diagram into a modified structure of product components; (**c**) and the FR/DP relations.

Here the authors want to demonstrate the practicability of the S_DC_ using an example of a customizable personal computer containing four stable groups of components, namely the Hard-disk (HD) units, Motherboards (MB), Central Processor Units (CPU), and Operating Systems (OS). These groups consist of compulsory optional components with volition rules, see feature diagram in Figure 5a. This feature diagram can be in the next step transformed into a PPc as depicted in Figure 5b.

For the purposes of comparing the two product structures, particular volition rules are applied. Component S3 in model A will be selected by an individual selectivity rule $\left(\begin{array}{c} 3\\ 1 \end{array}\right)$ (see Figure 5c; model A), while the same component in model B will be selected by an individual selectivity rule $\left(\begin{array}{c} 3\\ 2 \end{array}\right)$ (see Figure 5c; model B). Then, the number of all PPVs can be easily determined, as shown in Figure 5c. The number of PPVs equals 12 for the models.

When applying the complexity indicator S_DC_ to models A and B using matrices, the following design matrix values are obtained:

model A

$$S_{DC}=6\cdot ln6+6\cdot ln6+6\cdot ln6+6\cdot ln6+4\cdot ln4+4\cdot ln4+4\cdot ln4+12\cdot ln12=89.5\;\mathrm{nats},$$

model B

$$S_{DC}=6\cdot ln6+6\cdot ln6+6\cdot ln6+6\cdot ln6+8\cdot ln8+8\cdot ln8+8\cdot ln8+12\cdot ln12=122.7\;\mathrm{nats}.$$

Summarizing the findings of this realistic study, we can see the following practical implications of the assessment of product variety complexity through the use of an S_DC_ indicator. When assessing product variety complexity using the PPV indicator, the two models seem to be equally complex. However, in reality, model B is more complex than model A. To be specific, the difference in complexity between models A and B is 27.1%. Then, it is obviously justified to prioritize the proposed product variety complexity measure over the indicator based on the maximum number of possible product variants.

The purpose of this case study was to provide a validation of the proposed approach to estimate product variety complexity in terms of mass customization. Based on a randomly selected case study problem, it was shown that the proposed approach to quantifying variety-based complexity brings useful information as well as a new insight into this specific entropy-complexity nexus.

## 5. Conclusions

The presented results from computational experiments indicate promising practical utilization of the proposed approach to measure product variety complexity in a mass customization environment. It was shown and proved that the S_DC_ indicator better reflects product variety complexity than the total number of all possible product configurations. Moreover, it is also useful to mention that practically any model of initial components and related product configurations can be transformed into a design matrix, and product variety-induced complexity can be easily determined based on that data.

Empirically, it can be stated that the given approach is applicable to any possible product platform structure that consists of the common types of components, such as stable, optional, and compulsory optional ones. In cases, where the product platform consists of unusual type(s) of component(s), then the proposed procedure would need to be modified accordingly. However, this fact does not cause a significant problem in applying the proposed approach, since eventual changes in the nomenclature of product components can be relatively easily handled in the presented classification framework of product component types (see Section 3.2). Taking into consideration that the problem of variety-induced complexity is treated by other researchers from different viewpoints, the proposed method can serve as a complementary tool in this domain.

Further research could be focused on examining to what extent various types of optional components, such as voluntary, compulsory, and those designed by the customers, impact variety-induced complexity and related manufacturing complexity.

## Data Availability

Not applicable.
